# Supplementary material for: Characterization of Serum Cytokine Profiles of Patients with Active Lupus Nephritis
Source: Int J Mol Sci. 2023 Oct 4;24(19):14883. doi: 10.3390/ijms241914883 (PMC10573765; doi:10.3390/ijms241914883)
Supplement: Supplementary file 1 [file ijms-24-14883-s001.zip › ijms-2613733-supplementary.pdf]

Table S1. Cytokines serum levels over different timepoints.

CK	T0	T3	T6	p-value
**IL-37 (ng/ml)**	0.04 (0-0.22)	0.04 (0-0.11)	0 (0-0.06)	0.034
**BAFF (ng/ml)**	1.30 (0.81-2.46)	1.15 (0.89-1.91)	1.17 (0.99-3.65)	0.045
**IL-2 (pg/ml)**	0 (0-0.68)	0 (0-0.11)	0 (0-0.14)	0.801
**IL-10 (pg/ml)**	0 (0-12.7)	0 (0-4.27)	0 (0-4.0)	0.107
**IL-17 (pg/ml)**	1.03 (0-5.67)	0.25 (0-5.56)	0.13 (0-2-09)	0.618
**IL-18 (ng/ml)**	0.282 (0.174-0.389.5)	0.238 (0.148-0.301)	0.275 (0.173-0.407)	0.920

Table S1. Median (IQR) cytokines concentrations at baseline (T0), 3 months (T3), and 6 months (T6) timepoints and p-values referred to multiple comparisons for each cytokine (ANOVA test).
